# Patch Clamp: A Powerful Technique for Studying the Mechanism of Acupuncture

**DOI:** 10.1155/2012/534219

**Published:** 2012-10-22

**Authors:** D. Zhang

**Affiliations:** ^1^Department of Mechanics and Engineering Science, Fudan University, Shanghai 200433, China; ^2^Shanghai Research Center for Acupuncture and Meridians, Shanghai 201203, China

## Abstract

Cellular and molecular events can be investigated using electrophysiological techniques. In particular, the patch-clamp method provides detailed information. In addition, the patch-clamp technique has become a powerful method for investigating the mechanisms underlying the effects of acupuncture. In this paper, recent researches on how acupuncture might modulate electrophysiological responses in the central nervous system (CNS) and affect peripheral structures are reviewed.

## 1. Introduction

The therapeutic mechanisms of acupuncture are based on the theory of acupuncture and meridians in traditional Chinese medicine (TCM). How to use modern medical research methods to clarify the mechanisms of acupuncture is one of the important subjects of current basic research in TCM. Currently, the microstructure of human tissue, including the complex relationship between cells and the interstitium, and the laws of information transmission in the human body are understood. However, the essence of acupuncture-effect mechanisms is still unclear.

Cellular signal transduction theory is critical for understanding the mechanisms that explain the effects and actions of acupuncture. Cells constitute the basic units of living organisms, and the synergy between cells facilitates the various functions of the body. Communication between cells relies on receptors and ion channels in the cell membrane. Using patch-clamp technology, the electrical changes of cell membrane ion channels can be observed to elucidate the cellular mechanisms of acupuncture. As a well-developed cellular electrophysiological method [1, 2], the patch-clamp technique has set the stage for further studies of the mechanisms of ion channels, signal transduction, and the nerve transduction system. This method is now widely used in the basic and applied research of various disciplines, such as physiology and pharmacology [3]. The patch-clamp technique provides a fairly direct way to study the gating dynamics, permeability, and selectivity of ion channels in cell membranes. Acupuncture, which is based on meridian theory, can induce therapeutic effects throughout the entire body in a multiaction and multitarget fashion. These effects, as a type of biological information transfer, most likely involve cellular electrophysiological and biochemical changes similar to the effects of various drugs. The patch-clamp technique may deepen the study of acupuncture and clarify the underlying principles. The present paper reviews the application of patch-clamp technology in acupuncture research.

## 2. Study of Acupuncture-Induced Changes in the Central Nervous System 

### 2.1. Recordings from Isolated Neurons

Acupuncture is a viable and complementary treatment for relieving pain; the treatment involves the insertion of needles to a certain depth at specific acupuncture points (acupoints) followed by manipulations or electrical stimuli [4–7]. Despite the widespread use and confirmed efficacy of acupuncture for specific areas of pain management, the mechanism of acupuncture-induced analgesia remains unclear [8, 9]. This ambiguity occurs because studying the characteristics and regulatory function of the CNS network during acupuncture is rather complicated. 

Researchers use different pain models to investigate the role of neuronal membrane receptors in the sensation, transmission, and modulation of pain [10, 11]. Unlike the evaluation of the tail flick reaction, which involves behavioral functions, electrophysiological recordings of extracellular single-unit discharges are relatively pure indicators of spinal nociceptive activity during acupuncture [12]. The *in vitro* patch-clamp technique more precisely demonstrates the interactions between multiple receptors [13].

The mechanisms of acupuncture-induced analgesia have been investigated since the 1950s as a key project of the Natural Science Development Plan in China [14]. The results of the research from the group led by Professor Han for more than 20 years showed that cholecystokinin octapeptide (CCK-8) in the CNS functions as an antiopioid substrate and antagonized opioid- or EA-induced analgesia [15, 16]. First, extracellular single-unit recordings were made from spinal dorsal horn wide dynamic range neurons in spinal-transected, urethane-anesthetized rats. EA at the ST-36 and SP-6 acupoints effectively suppressed the unit discharges elicited by noxious electrical stimulation of the hind paw. Local spinal superfusion with CCK-8 (10 ng/15 mL) attenuated, whereas the CCK-selective antagonists L365 and 260 (2.5 *μ*g/15 mL) enhanced the EA effect [17–19]. All three types of opioid receptors (*μ*, *δ*, and *κ*) in the spinal cord of rats play important roles in mediating the analgesia induced by EA [20]. Second, the sites of opioid-CCK interaction were precisely localized using the whole-cell patch-clamp technique on acutely dissociated dorsal root ganglion (DRG) neurons from rats. The dose-dependent inhibition relationship indicated that CCK-8 antagonized the *μ*- or *κ*-opioid-receptor-mediated depressant effect on the voltage-gated calcium current. The CCK receptors were presynaptically coexpressed with the opioid receptors in the same neuron [21, 22]. This finding could explain why the effect of EA subsided after prolonged (6 h) EA stimulation, suggesting the development of tolerance to EA [23]. Because CCK-8 has been shown to possess potent antiopioid activity at the spinal level, a blockade of the spinal CCK effect would be expected to potentiate EA-induced analgesia, which is known to be opioid mediated [24].

In neuropathic pain, adenosine 5′-triphosphate disodium- (ATP-) gated P2X receptors, especially the P2X3 subtype, play an important role in the transmission of pain signals [25, 26]. For electrophysiology recordings, a chronic constriction injury (CCI) model of neuropathic pain was induced by tying four ligature knots proximal to the sciatic trifurcation of the right nerve in rats [27]. After the CCI operation, EA was applied at the ST-36 and GB-34 acupoints for 30 min daily for one week. The DRG neurons were acutely isolated from the ganglia after enzymatic digestion, and a conventional whole-cell patch clamp was performed. As P2X3-receptor agonists, both ATP and *α*,*β*-methylene-ATP (*α*,*β*-meATP) evoked similar fast desensitizing inward currents in the DRG neurons. The amplitudes of the currents in the CCI group were larger than in the sham group and were significantly attenuated by the EA treatment (no differences between ipsilateral and contralateral). EA may induce an apparent analgesic effect by decreasing the expression of and inhibiting P2X3 receptors in the DRG neurons of rats with CCI [27].

Visceral pain is difficult to be localized and easily transferred to cutaneous structures. Some natural stimuli are clearly associated with multisourced pain from the viscera, such as hollow organ distention, muscle spasm, ischemia, and inflammation [28]. EA is clinically used for the treatment of chronic visceral hyperalgesia (CVH). It is difficult to explain with the classical description in which there are segregated routes in the CNS for visceral and somatic inputs [29, 30]. Using the patch-clamp technique, the inhibitory effect of acupuncture on visceral sensory neuron excitability can be observed directly, which supports the interpretation of disorientated visceral pain and referred pain.

To investigate the effect of EA on colon-specific DRG neurons in rats with CVH and to clarify the interactions between somatic and visceral nociceptive inputs in the spinal dorsal horn, electrophysiological measurements were made [31]. Before perforated whole-cell recordings from DRG neurons were performed, a CVH model was induced by intracolonic injection of acetic acid in 10-day-old rats. EA was applied bilaterally at ST-36 in the hind limbs. The acutely dissociated colon-specific DRG neurons were prelabeled by injection of 1,1′-dioleyl-3,3,3′,3-tetramethylindocarbocyanine methanesulfonate (DiI) into the colon wall 10 days before acupuncture. The resting potentials and action potentials showed the passive and active membrane properties. The recordings from the CVH group displayed enhanced excitability of the colon DRG neurons; this enhancement was significantly suppressed in recordings from the EA group after treatment. Furthermore, the *in vitro* application of the *μ*-opioid receptor agonist (D-Ala (2), N-MePhe (4), and Gly (5)-Ol) enkephalin (DAMGO) mimicked the EA-mediated suppression. These findings revealed the endogenous opioid-related analgesic effects at the cellular level during EA treatment of visceral pain in functional gastrointestinal disorders.

### 2.2. Recording from Neurons in Living Slices

Patch-clamp recordings from brain slices have been utilized to analyze CNS function since the 1980s [32–34]. By applying the patch-clamp technique to brain slices, which constitute a simple network system *in vitro*, the effects of acupuncture on target cells can be directly observed because the blood-brain barrier is bypassed. With the proper conditions (increased oxygen supply and modified artificial cerebrospinal fluid (aCSF) content), the metabolic and biological properties of the brain slices can be maintained *in vitro* for a couple of hours [35], which forms an ideal system for recording electrophysiological activities. This system facilitates the investigation of the channels of the CNS and their physiological functions in neuroanatomical circuits.

An imbalance between the excitation and inhibition of gamma-aminobutyric acidergic (GABAergic) neurons caused by ischemia-induced neuron dysfunction has been suggested as an alternative mechanism for neuronal excitotoxicity [36]. Stimulation of certain acupoints, such as Baihui (GV-20), has been found to improve the outcome of ischemic stroke [37, 38]. The function of GABAergic neurons can be evaluated based on their intrinsic properties as well as their response to excitatory synaptic inputs from other neurons [39]. EA was applied to FVB-Tg (Gad GFP) 4570 Swn/J mice at DV-20 for 20 min, twice daily for a week. Cortical slices (400 *μ*m) were sectioned from the acupuncture and sham groups and superfused with the aCSF oxygenated at 31°C. *In vitro* ischemia was generated by reducing the perfusion rate from 2 mL/min to 0.2 mL/min for 6 min. Ischemia generally induces increases in the threshold potential and absolute refractory periods of firing spikes in neurons that are measured in the whole-cell recording mode with sequential spikes induced by depolarizing current pulses; acupuncture prevented these increases. Meanwhile, spontaneous excitatory postsynaptic currents (sEPSCs) were recorded in the GFP-labeled GABAergic neurons. The impairment of active transmissions between neurons as a result of ischemia was significantly prevented with acupuncture. Acupuncture improves ischemic stroke via preventing this dysfunction by targeting voltage-gated sodium channels [40]. Modulation of GABAergic activity by inhibition of the GABA reuptake transporter GAT1 has also been suggested [41]. As discussed above, EA leads to an elevated release of endorphins [15]; it was also demonstrated that activation of *δ*-opioid receptors in the periaqueductal gray results in inhibition of GAT1-mediated current as measured in whole-cell recordings.

### 2.3. Recording from Neurons *In Vivo*

The *in vivo* patch-clamp technique is mainly used to study the characteristics and mechanisms of the sensory system in response to environmental stimuli [42]. The protocol for the blind patch-clamp method was first introduced *in vivo* for whole-cell recording. Later, the success rate was significantly improved by two-photon targeted patching (TPTP), in which the patch clamp is performed under direct visual control by imaging the fluorescence with two-photon microscopy (2PM) [34, 43]. The visualized operation of targeted patching allows a depth of 0.5 mm, which is approximately half of a young rat's cerebral cortex. In addition to the progress in labeling, the application of *in vivo* patch clamp has been broadened. Compared with *in vitro* isolated neurons, *in vivo* measurements on brain or spinal cord slices have a greater advantage: one can obtain not only the final outputs but also the subthreshold responses. With this information, analyzing the interaction between excitatory and inhibitory synaptic inputs activated by cutaneous stimulation becomes possible [44, 45]. The method provides an effective means for real-time observation of sensory neuronal transmission in the central and peripheral nervous systems.

The substantia gelatinosa (SG, lamina II of the spinal cord) may be a major site for the modification and integration of noxious sensation. A chronic pain model was established by complete Freund's adjuvant- (CFA-) induced inflammation in the right hind paw of rats. A lumbar laminectomy was performed on an anaesthetized rat at the L4 or L5 level. The surface of the spinal cord was exposed by removal of the pia-arachnoid membrane and was irrigated with oxygenized Krebs solutions at 38°C. *In vivo* whole-cell voltage-clamp recordings were applied during acupuncture to the SG neurons, which were morphologically identified by injection of neurobiotin [46]. Spontaneous inhibitory postsynaptic currents (sIPSCs, *V*_*H*_ = 0 mV) and sEPSCs (*V*_*H*_ = − 70 mV) were recorded from the same neurons by altering the holding potentials. In the CFA model group, sEPSCs with higher magnitudes were observed in the majority of SG neurons. The application of EA to the contralateral ST-36 point slightly decreased the amplitude and frequency of the sEPSCs. In contrast, a larger amplitude and higher frequency of sIPSCs were elicited with acupuncture frequencies from 2 to 10 Hz. This *in vivo* patch-clamp recording technique permits functional analyses of modality-dependent synaptic responses evoked by acupuncture. Therefore, the behavioral changes at the single-cell level can be explained with more certainty [47].

## 3. Study of the Mechanism of Peripheral ****Signaling Initiation in Acupoints and Related Structures Using Patch

### 3.1. Recording on Acupoint Enriched with Mast Cells

As the basic elements in meridian structure, acupoints are suggested to serve as the initiating points for acupuncture treatments [48]. Despite considerable efforts in probing the anatomy of acupoints, the structural characterization of acupoints remains elusive. Many methods have been employed to research the specificity of acupoints [49]. Compared with the surrounding tissue, acupoints were found to be richer in connective tissue structures [50], mast cells [51, 52], capillaries, and nerve endings [53]. In addition, significantly elevated concentrations of Ca, Fe, Cu, and Zn were reported [54]. The densities of mast cells in the acupoints of both skin and muscle tissues were approximately 50% higher than those in sham points [55]. Some researchers [56–58] have measured the sensitivities of different receptors on specific cells as a breakthrough point to explore the mechanism of peripheral signaling initiation in acupoints, including related structures, at the cellular and molecular levels.

Sensitivity to mechanical stimulation is an inherent property of many tissues and cells. The patch-clamp technique can directly study the responses of mechanosensitive (MS) ion channels [59], which are physiologically implicated in the processes of acupuncture manipulations in acupoints [60]. Along with the attenuation of analgesia or the regulatory effect from acupuncture at ST-36, pretreatment with collagenase significantly counteracted the acupuncture-induced increase in the number of degranulated mast cells [61, 62]. These results suggest that collagen fibers in the subcutaneous connective tissue act as a transduction medium for mechanical signals between manual acupuncture stimulation and mast cells. What is the underlying mechanism that triggers mast cell degranulation after the mechanical signals are received? Ion channels that are selective for K^+^, Na^+^, and Ca^2+^, TREK and TRAAK [63], TRPV2 [64], and TRPV4 [65] are typical proteins that mediate mechanosensitive cation movements across the lipid membrane. Cl^−^ channels are the primary mechanosensitive anion channels that play an important role in volume regulation [66].

Research on MS ion channels and the proteins that might play roles in the acupuncture effect was conducted at the cellular level using a human leukemia mast cell line (HMC-1) [56, 67]. In the whole-cell patch-clamp configuration, the mechanical stress applied to a cell membrane by hydrostatic pressure was increased (−30 to −90 cm H_2_O applied via a patch pipette). This stress induced an inward-directed current along with cell degranulation [56]. The activation phenomenon was significantly blocked by Ruthenium Red (RuR), an inhibitor of the transient receptor potential vanilloid (TRPV) channel family, or by SKF96365, an inhibitor specific for TRPV2 [68]. The TRPV2-mediated current induced by mechanical stress exhibited a relatively low selectivity ratio (approximately 4) for divalent versus monovalent cations, as calculated from the reversal potential. Single-channel analysis in excised outside-out patches revealed strong outward rectifying, RuR- and SKF-insensitive events. Another component of the currents displayed single-channel events with chord conductance of 55 pS at positive potentials; this component strongly depended on Cl^−^ concentration, indicating that this current component may be mediated by Cl^−^ channels, possibly from the CLC family [67]. Further, the whole-cell current of mast cells was recorded *in situ* in connective tissue slices obtained from the ST-36 acupoint in rats. The resulting current to pressure gradient exhibited a similar outward rectification as for the activation of HMC-1 [57, 67]. Given that acupuncture-induced mast cell degranulation can be correlated with treatment effects, TRPV2 and Cl^−^ channels might be input receptors that contribute to the transduction process of the mechanical signal from acupuncture stimulations.

### 3.2. Recordings of Acupoint-Related Structures

As widely questioned in histological studies, the primo vascular system was first introduced as the anatomical structure of acupuncture meridians by Kim [69] and recently rediscovered in most tissues of the body [70]. As a semitranslucent thread-like structure, the PVS is composed of primo vessels (PV; Bonghan ducts) and primo nodes (PN; Bonghan corpuscles). The PNs were reported to contain cells such as mast cells, macrophages, and basophils [71]. The PNs were identified using confocal laser scanning microscopy in the epineurium along the rat sciatic nerve after subcutaneous injection of fluorescent nanoparticles at ST-36 [72]. Considering the close relationship between the peripheral nervous system and acupuncture treatment, further studying the primo vascular system to uncover its cellular properties may elucidate the mechanisms underlying acupuncture. Whole-cell recordings were performed on cells of a PN slice (200 *μ*m in thickness) isolated from the surface of abdominal organs in rats. The small round PN cells had a low resting membrane potential (−39 mV) and spontaneous activities [73, 74]. Of the four types of IV relationships and kinetics, most (69%) cells were type I and showed outward rectification. In current-clamp conditions, tetraethylammonium (TEA) dose dependently depolarized the membrane potential with an increase in input resistance [75]. The TEA-sensitive current with limited selectivity to K^+^ contributed to the resting membrane potential of these cells.

## 4. Prospects

The patch-clamp technique allows for the study of the properties of single channels expressed in specific cell membranes; progress has also occurred in research on the mechanisms of acupuncture. However, the depth (and breadth) is insufficient for a clear understanding of the processes that occur in acupoints and along meridians. By applying specific agonists, antagonists, and allosteric modulators to different parts of the membrane (inside or outside) at different times with fast perfusion, differentiated ionic currents through single channels can be recorded. These currents, together with the dynamic properties of the receptors, may help to demonstrate the existence and function of the detected receptor. 

Based on the technical potential of the patch-clamp method, the use of serum from acupunctured animals or humans [76] as a perfusate has been proposed to explore the mechanisms of acupuncture with regards to cellular electrophysiology. The serum may contain substances that act on targets such as cells or molecules in *in vivo* or *in vitro* tissues [77–79]. It will be informative to analyze the gating, ion selectivity, and regulation from single-channel currents before and after stimulation with serum. Further trials are required that vary the sampling time and method to determine the dilution and preservation of the serum and the involvement of the active ingredients from the reaction system. The application of serum to patch-clamp techniques requires further trials. 

Additionally, by using *in vivo* patch-clamp technology, the functions and relationships of intracerebral nuclei or spinal cords can also be observed during acupuncture; thus, the acupuncture pathway and the signal transduction process can be explored. Compared with investigations using patch clamp on isolated cells, there are only a few reports of *in vivo* studies because there are still difficulties in controlling factors, such as the mechanical fixation, anesthesia, and physiological interference of animals.

The application of electrophysiological techniques to study the mechanisms of acupuncture at the molecular level first requires the establishment of an animal model corresponding to the disease at the organ level. Then, single cells must be obtained from crucial organs or acupoints using cell separation and cultivation. Finally, cellular models must be built from different organs under pathological circumstances. By combining that information with specific agonists and antagonists, it is possible to determine the pathway of electrophysiological signal changes caused by acupuncture and to preliminarily explore the mechanisms of disease at the molecular level.

Using modern measurement techniques to study the mechanisms of acupuncture is one of the most important demonstrations of the modernization of Chinese medicine. The patch clamp can be combined with additional recordings to increase the analysis capabilities: with fluorescence measurement [80], the technique can be used to study the relationships between intracellular calcium concentration and secretion events; with the single cell reverse transcriptase-polymerase chain reaction (PCR) technique [81], patch clamp can be used to interpret why different cells of similar subtypes have different electrical activities. In addition, the patch-clamp technique can also reveal the pathways of intracellular information transmission and cell growth processes and even the complex interactions of central synapses. We believe that in the near future, patch-clamp methods will significantly contribute to unraveling the mystery of the mechanisms of acupuncture.
